# Midterm Experience of Ipsilateral Axillary-Axillary Arteriovenous Loop Graft as Tertiary Access for Haemodialysis

**DOI:** 10.1155/2014/908738

**Published:** 2014-03-23

**Authors:** J. P. Hunter, M. L. Nicholson

**Affiliations:** Transplant Group, Department of Infection, Immunity and Inflammation, University of Leicester, Leicester General Hospital, Gwendolen Road, Leicester LE5 4PW, UK

## Abstract

*Objectives*. To present a series of ipsilateral axillary artery to axillary vein loop arm grafts as an alternative vascular access procedure for haemodialysis in patients with difficult access. *Design*. Retrospective case series. *Methods*. Patients who underwent an axillary loop arteriovenous graft from September 2009 to September 2012 were included. Preoperative venous imaging to exclude central venous stenosis and to image arm/axillary veins was performed. A cuffed PTFE graft was anastomosed to the distal axillary artery and axillary vein and looped on the arm. *Results*. 25 procedures were performed on 22 patients. Median age was 51 years, with 9 males and 13 females. Median number of previous access procedures was 3 (range 0–7). Median followup was 16.4 months (range 1–35). At 3 months and 1 year, the primary and secondary patency rates were 70% and 72% and 36% and 37%, respectively. There were 11 radiological interventions in 6 grafts including 5 angioplasties and 6 thrombectomies. There were 19 surgical procedures in 10 grafts, including thrombectomy, revision, repair for bleeding, and excision. *Conclusions*. Our series demonstrates that the axillary loop arm graft yields acceptable early patency rates in a complex group of patients but to maintain graft patency required high rates of surgical and radiological intervention, in particular graft thrombectomy.

## 1. Introduction

Arteriovenous fistula (AVF) is the recommended modality of access for patients on haemodialysis for end-stage renal failure. Guidelines from the National Kidney Foundation (KDOQI) suggest that all AVF options should be exhausted before resorting to central venous access catheters [1]. In the majority of patients, arm fistulas will be all that is required; however, there is a cohort of patients in whom vascular access is problematic who require more complicated access procedures. Once all native arm and forearm AVF have failed, then the options are limited and are broadly, either a graft involving the axillary or central vessels, or a lower limb arteriovenous access procedure. Lower limb access is typically the last resort and has high infection rates, risk of limb loss and potentially compromises the iliac arteries for future transplants; therefore an upper limb synthetic graft should be the next procedure of choice [1–4]. There are three groups of patients that have benefitted from axillary grafts to date: first, those noted above in whom the upper limb vein options are exhausted, usually due to thrombosis from previous AVF; second, those with severe vascular steal syndrome from a conventional arm AVF; and third, those patients with small brachial vessels, usually due to renal osteodystrophy from long-standing renal failure. Early axillary-axillary grafts for haemodialysis used a bovine carotid artery as the conduit [5]. Since then a number of procedures have been described including both ipsilateral loop grafts and contralateral procedures such as the axillary-axillary necklace graft [6–9]. Although the original axillary access operation was described over 35 years ago, it is still an uncommonly performed procedure and there are no UK series with outcomes of ipsilateral axillary loop grafts. We have previously described the ipsilateral axillary loop graft as a tertiary access procedure for haemodialysis and this manuscript details our experience of the first 25 cases [10]. The literature on vascular access options for haemodialysis in complex patients is limited. We detail one of the largest series of upper limb loop grafts for haemodialysis access in patients with failed arm fistulas. In doing so, we provide an additional vascular access procedure, in the form of an axillary loop graft, as an option for patients with complex vascular access. The axillary loop graft avoids central venous catheter use and delays the need for lower limb procedures.

## 2. Methods

A consecutive series of patients who underwent an axillary loop graft for haemodialysis access between September 2009 and September 2012 were included in the study. All patients were initially assessed through the consultant-led vascular access service at our institution and were investigated preoperatively with contrast venogram to identify the venous anatomy and exclude any central venous stenosis or obstruction. Venography was used ahead of duplex ultrasound because it has the combined advantage of illustrating all of the arm vessels, ensuring that none were suitable for AVF formation, and imaging the central veins for stenosis or obstruction. Venography was performed by a single consultant radiologist who reported the veins as poor, moderate, or good, but without a formal measurement; however, poor was considered less than 3 mm in diameter and those patients were considered unsuitable and excluded. The arterial sufficiency was assessed clinically and further investigations into the arterial anatomy were at the discretion of the named consultant. All patients had either exhausted optimal AVF sites or had unsuitable venous anatomy for a more distal AVF. The indications for axillary loop graft were exhaustion of forearm and arm venous options; multiple failed brachial artery fistulas (which could be due to a high branching of the radial artery and consequently a small brachial artery); steal syndrome from previous brachial artery fistula; or constitutionally small veins.

### 2.1. Surgical Procedure

**Figure 1:**
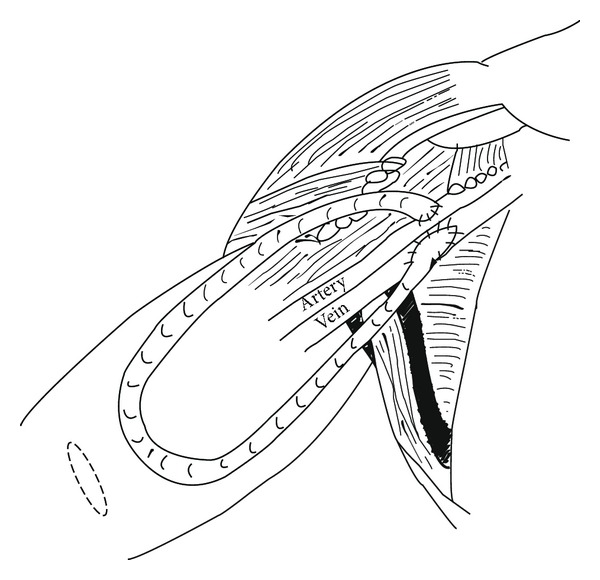
Illustration of axillary artery to axillary vein loop graft. Teres major muscle is darkened to demonstrate more clearly the anatomical location of the anastomoses. The transverse incision on the arm, illustrated by broken lined ellipse, facilitates tunneling of the graft.

The procedure was carried out under general anaesthesia or regional anaesthetic block and performed or supervised by one of two consultant transplant surgeons and has been previously described [10]. Prophylactic antibiotics (Teicoplanin 400 mg IV) were given at induction. The distal axillary artery and axillary vein were exposed and controlled via a transverse incision in the axilla. An end to side anastomosis was performed between the axillary vein and graft using a cuffed PTFE 6 mm (polytetrafluoroethylene) graft with a venous funnel which was trimmed to size (Bard Limited, Crawley, UK) using 6/0 Gore-Tex (W.L. Gore & Associates, Inc., Arizona, USA) suture. A transverse incision was then made proximal to the antecubital fossa to allow tunnelling of the graft to fashion a loop. An end to side anastomosis between the distal axillary artery and graft was then fashioned using a 6/0 Gore-Tex suture. The fascial layers were then closed with a 3/0 Vicryl suture and the skin closed sing 4/0 Prolene sutures (Figure 1). Postoperatively, clinical observations (pulse, blood pressure, respiratory rate, and oxygen saturations) and fistula-specific observations (thrill and bruit) were performed. Two further doses of antibiotics (Teicoplanin 400 mg IV) were administered in the following 24 hours. After discharge, the patients had their graft assessed during dialysis sessions by nursing staff to ensure patency and were reviewed at 1 month in the vascular access clinic to decide whether the graft was suitable for needling.

### 2.2. Dialysis Surveillance

Surveillance was only carried out in our institution after December 2010 and included graft blood flow (mL/min) and urea reduction ratio (%). If there was a reduction in either value on two separate occasions, a Duplex Ultrasound scan was performed. Duplex ultrasound was also performed as the first-line investigation for graft dysfunction. Intervention to retain patency was discussed at a vascular access multidisciplinary meeting, with surgeons, nephrologists, and radiologists present.

## 3. Results

### 3.1. Demographics

Twenty-five axillary loop grafts were performed in 22 patients during the study period and were included for analysis. The centre performs an average of 300 arteriovenous access procedures per annum with axillary grafts accounting for about 3% of the total. There were 9 males and 13 females with a median age of 51 years (range 23–81). The median number of previous vascular access procedures was 3 (range 0–7) and this included 5 patients who had undergone a total of 7 brachioaxillary grafts. Comorbidity data are illustrated in Table 1. The indications for an axillary graft were exhausted arm and forearm veins or multiple failed brachial AVF (18); severe steal syndrome from previous brachial artery AVF (1); and constitutionally small veins (3). One patient had an axillary graft as their initial access procedure as all arm veins were inadequate. All patients who were dialysing at the time of graft creation had previous internal jugular vein catheters and some also had previous subclavian venous catheters. The median followup was 16.4 months (range 1–35). Fifteen grafts were left sided and 10 were right sided.

### 3.2. Dialysis

Median time to first dialysis was 43 days (range 11–95). Eight grafts were never used for dialysis. Three of the eight grafts were functioning at the time of analysis and of those 2 patients were transplanted and 1 was predialysis. Of the remaining 5 grafts, 3 failed within the first week and can be classed as primary failures, one was excised on day 49 due to infection, and one underwent thrombectomy and revision on day 37. Flow rates on dialysis, through the machine, for the functioning grafts were good ranging between 500 and 800 mL/min.

### 3.3. Patency Rates

**Figure 2:**
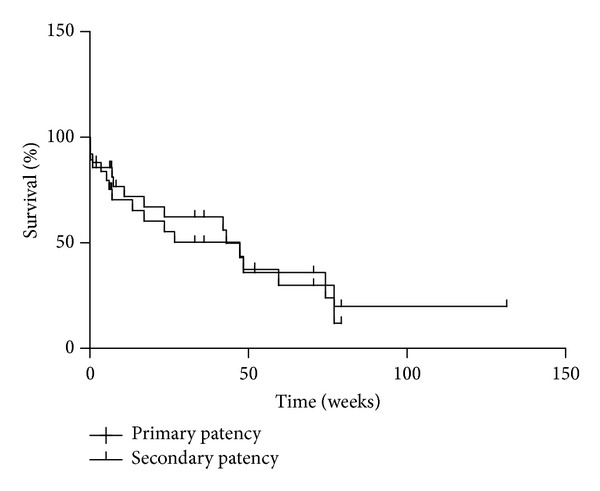
Kaplan-Meier survival curves illustrating primary and secondary patency of axillary loop grafts over time (weeks). The number of grafts at risk and curves with standard error can be found in Supplementary Figure 1 in Supplementary Material available onlineat http://dx.doi.org/10.1155/2014/908738.

At 1 and 3 months, the primary patency rates were 84% and 70%, respectively, and the secondary patency rates were 86% and 72%. At 6 months, the primary and secondary patency rates were 50% and 62%, respectively, and at 1 year they were 36% and 37%, respectively. Kaplan Meier curves illustrating the primary and secondary patency are illustrated in Figure 2. The 7 patients with previous brachioaxillary grafts underwent 8 axillary loop grafts, 3 of which had good secondary patency (539, 494, and 294 days) and 5 of which had poor patency.

### 3.4. Intervention

There were 11 radiological interventions in 6 grafts. Five were angioplasty of a stenosis, all of which successfully retained graft patency. The remaining 6 interventions were graft thrombectomy, which were performed using the AngioJet thrombolysis catheters, 4 of which were successful and 2 required surgical intervention the following day. One thrombectomy resulted in a brachial embolus requiring surgical intervention and the ischaemic arm was salvaged but the graft was lost. Nineteen surgical procedures were undertaken in 10 grafts. There were 10 thrombectomies in 5 grafts and 3 of these grafts required surgical revision. The three graft revisions were performed as a result of graft occlusion and followed failure of surgical thrombectomy. Surgical thrombectomy was undertaken mechanically with a Fogarty catheter, and if it was unsuccessful, the venous anastomosis was revised. Four grafts required intervention for bleeding, 2 from needling defects and 2 from pseudoaneurysm. Four grafts were excised due to infection.

### 3.5. Complications

Postoperative arm swelling was the most frequent complication occurring in 5 patients. Four patients were treated for wound infection/cellulitis and 2 patients had subcutaneous collections. One patient had a clinically significant steal syndrome and had an angioplasty of a brachial artery stenosis. There were no postoperative deaths; however, 3 patients died during the study period. The cause of death was unrelated to the graft and all 3 patients had a functioning graft being used for dialysis at the time of death. Five grafts failed prior to being used for dialysis: one graft was excised at day 49 due to infection; 3 grafts failed within 1 week of which two underwent revision and one patient refused further surgery; one failed at day 52 following surgical thrombectomy and revision (without radiological intervention); one failed at day 76 following surgical revision.

## 4. Discussion

Patients with multiple failed arteriovenous fistulas requiring complex tertiary access procedures are increasingly commonplace. Many of these patients have failed peritoneal dialysis and are either unfit for transplantation or highly sensitised from previous transplants. This study demonstrates that the axillary loop graft yields secondary patency rates at 3 months of 72% that are above the National Kidney Foundation recommendation of 70% [1]. However, the secondary patency rate at 1 year of 37% was lower than that of previously published studies, although none of these include loop grafts. Interestingly the primary and secondary patency rates at 1 year were similar at 36% and 37%, respectively. This suggests that there are a proportion of grafts that stay complication-free without neointimal hyperplasia and as a result remain widely patent, although the reason for this is unclear.

Axillary procedures are well described but uncommonly performed, and since the first procedure, described by Manning et al. in 1975, there have been few case series described [5]. The largest is Jean-Baptiste and colleagues' French series of 27 patients, which shows favourable primary and secondary patency rates at 1 year of 51% and 80%, respectively [7]. The patency rates that they achieve are more comparable with those found in grafts used as primary procedures. In their series, 8 (30%) patients had an axillary graft as an initial access procedure. Literature from the United States where PTFE grafts are commonly used ahead of native veins yields similar patency rates with values up to 85%. Jean-Baptiste et al. conclude that axillary loop grafts are a more preferable option than lower limb vascular access, a contention this paper fully supports. However, in contrast to our procedure in which a loop is created in the arm and involves two small axillary incisions, their procedure involves 3 incisions including an infraclavicular incision, the graft is tunnelled in the chest, and the arterial anastomosis is more proximal. There are merits to both approaches and the details above merely demonstrate the difference in technique. Morsy and colleagues have described a series of 18 patients with axillary-axillary necklace grafts, which is a contralateral graft that is tunnelled across the chest anterior to the upper third of the sternum. They also demonstrate favourable results with a primary patency rate of 66.7% after a median followup of 19.7 months and a secondary patency rate of 88.9% after a median followup of 14.9 months [6]. This is in contrast to some of their previously published data on ePTFE grafts (not axillary grafts) with a 12-month secondary patency rate of 68% [11]. Indeed the patency rates of ePTFE grafts vary widely in the literature with values ranging from 38 to 85% [12–16]. There are other upper limb options such as the interarterial axillary-axillary graft of which there is a case series with primary and secondary patency of 90% and 93% at six months. However, these patients had completely exhausted venous options, and thus the risks of an artery-to-artery graft were worthwhile [17]. A variety of lower limb procedures have been described and use of the saphenous vein as a conduit has rather lost favour due to the high rates of failure and need for further intervention [18–22].

The authors acknowledge the limitations of retrospective data but present a series of axillary grafts with a patient number comparable to that of previously published series, in a complex group of patients. The lower than expected patency rates at 1 year are likely to be multifactorial. The number of previous access procedures highlights the complexity of the patient population, with some patients having undergone 7 previous procedures excluding central venous catheters. Arteriovenous fistula surgery yields a diminishing return with each procedure performed and it is thus not surprising that patients unable to sustain access using native arm veins have relatively poor outcomes with grafts [23, 24]. Moreover, in contrast to previous studies, only one patient in our series underwent a graft as an initial procedure. The patient had unsuitable arm veins and had a rapidly deteriorating glomerular filtration rate, which was 10 mL/min at the time of the procedure. The justification for the procedure is that the KDOQI guidelines state that patients should have definitive vascular access at the initiation of dialysis and avoid temporary central venous catheters. Indeed in the UK, the hospital trust is financially penalised per dialysis session, for each patient that dialyses via a central venous catheter. The graft was not used in this particular patient as he received a deceased donor transplant within 1 month of the graft placement. The lack of radiological intervention and surveillance, which was crucial for the excellent patency rates described in previous studies, is also likely to have a bearing on the overall patency [6]. Indeed, prior to December 2010, there was no radiological intervention in our centre. This resulted in the high number of surgical thrombectomy procedures detailed above and the loss of grafts due to untreated venous stenosis. The institution of a surveillance programme to highlight dysfunctioning grafts and their subsequent imaging and intervention prior to thrombosis should reduce the need for such frequent surgical intervention, which undoubtedly increase the risk of graft infection and further complications.

Patient selection, in relation to vein quality and diameter, is also important, although early failures lower the overall patency rates that should not discourage the surgeon from attempting a graft. Such patients will have high rates of failure, illustrated by the two early failures in our series, but the authors feel that as long as the patient is well informed about the risk of failure, it is acceptable to attempt such procedures and, in so doing, avoid the need for lower limb procedures and long-term central venous dialysis catheters. The authors acknowledge that in the presence of small axillary vessels, the infraclavicular incision may be more beneficial than axillary approach, as the axillary approach may limit the proximal vein exposure. Four grafts were excised due to infection and/or pseudoaneurysm formation. Minimizing this risk rotation of needling sites along the graft is suggested but unfortunately the so-called buttonholing technique is favoured and altering this behaviour to reduce infection risk is difficult. Early graft failures are inevitable, regardless of surgical technique and are as a result of small veins, atherosclerotic and calcified arteries, or a combination thereof. The complication rate overall was comparable to previous studies [6, 7]. Graft thrombosis was the most frequent complication with 16 episodes in 6 grafts. It has been suggested that loop grafts are more susceptible to intimal hyperplasia at the venous anastomosis, due to the high flow rates and orientation of the venous anastomosis, and are at greater risk of rupture. Although grafts have been surgically corrected for bleeding, we are yet to encounter graft rupture at the venous anastomosis. The 6 mm funnelled (cuffed) graft permits a large, appropriately aligned venous anastomosis and is fashioned to reduce the rate of venous stenosis. However, there has been suggestion contrary to this; that is, the venous funnel (cuff) leads to turbulent blood flow, which stimulates intimal hyperplasia and stenosis. The evidence is far from conclusive, but of the available literature comparing cuffed and uncuffed grafts, there are a lower rate of thrombosis and greater patency in favour of the cuffed graft [25, 26]. Finally, the configuration of a loop may present more difficulty in completing a thrombectomy without leaving a small amount of residual thrombus at the venous anastomosis. This would then lead to early rethrombosis and may explain the graft losses in our series following surgical thrombectomy. The loop configuration on the arm may also be susceptible to kinking with the arm in certain positions, although the authors have no objective evidence to support this conjecture. The infection rate of 16% is slightly higher than some of the US data; however, when including thigh loop grafts, this value is quoted as high as 35%. Our patient population is not comparable with those having a graft as an initial procedure, as the multiple comorbidities and state of relative immunosuppression of the chronic haemodialysis patient render these patients at high risk of infection.

The main advantage of an axillary loop graft is that it is a procedure for patients with difficult access that utilises the ipsilateral artery and vein and as such preserves the dominant arm. Furthermore, it potentially permits another graft on the unused side if the initial graft fails and avoids, for as long as possible, the need for a lower limb procedure. Indeed three patients within the study have undergone bilateral grafts. Although only a minor advantage, cosmetically the graft is more discrete than the ones tunnelled in the chest and neither is the graft at risk of damage if a sternotomy were required for thoracic access.

We have described our series of 25 axillary loop grafts and consequently present an alternative upper limb procedure in complex patients with exhausted conventional vascular access or inadequately sized arm vessels for brachial AVF formation.

## Supplementary Material

Supplementary figure 1. shows Kaplan-Meier survival curves illustrating primary and secondary patency of axillary loop grafts over time (weeks) with error bars showing standard error. The number of grafts at risk is also shown.

## Figures and Tables

**Table 1:** Demographic data for the 22 patients included in the study. The number (*n*) of patients affected is displayed in the right hand column and the % affected is in parentheses.

	Number of patients, *n* (%)
Diabetes	7 (31%)
Hypertension	19 (86%)
Dyslipidemia	11 (50%)
Warfarin therapy	6 (27%)
Antiplatelet therapy	8 (36%)
Previous central venous catheter	20 (91%)
