# Supplementary material for: Midterm Experience of Ipsilateral Axillary-Axillary Arteriovenous Loop Graft as Tertiary Access for Haemodialysis
Source: J Transplant. 2014 Mar 23;2014:908738. doi: 10.1155/2014/908738 (PMC3981058; doi:10.1155/2014/908738)
Supplement: Supplementary file 1 — Supplementary figure 1. shows Kaplan-Meier survival curves illustrating primary and secondary patency of axillary loop grafts over time (weeks) with error bars showing standard error. The number of grafts at risk is also shown. [file 908738.f1.docx]

Kaplan-Meier survival curves illustrating primary and secondary patency of axillary loop grafts over time (weeks) with standard error.

|  | primary patency | secondary patency |
| --- | --- | --- |
| 0.000 | 25 | 28 |
| 1.000 | 25 | 28 |
| 6.000 | 23 | 25 |
| 14.000 | 22 | 24 |
| 25.000 | 21 |  |
| 37.000 | 20 |  |
| 43.000 | 19 |  |
| 44.000 | 18 | 22 |
| 45.000 | 17 | 21 |
| 48.000 | 16 | 20 |
| 49.000 | 15 | 19 |
| 52.000 |  | 18 |
| 58.000 |  | 17 |
| 76.000 |  | 16 |
| 94.000 | 14 |  |
| 120.000 | 13 | 15 |
| 165.000 | 12 | 14 |
| 187.000 | 11 |  |
| 232.000 | 10 | 13 |
| 252.000 | 9 | 12 |
| 294.000 |  | 10 |
| 301.000 |  | 9 |
| 331.000 | 7 | 8 |
| 339.000 | 6 | 7 |
| 364.000 | 5 | 6 |
| 417.000 |  | 5 |
| 494.000 | 4 | 4 |
| 520.000 | 3 |  |
| 539.000 | 2 | 3 |
| 555.000 | 1 | 2 |
| 920.000 |  | 1 |

Number of grafts at risk
